# Reversed and increased functional connectivity in non-REM sleep suggests an altered rather than reduced state of consciousness relative to wake

**DOI:** 10.1038/s41598-021-91211-5

**Published:** 2021-06-07

**Authors:** Evan Houldin, Zhuo Fang, Laura B. Ray, Bobby Stojanoski, Adrian M. Owen, Stuart M. Fogel

**Affiliations:** 1 Brain & Mind Institute, Western Interdisciplinary Research Building, Western University, London, N6A 5B7 Canada; 2 Department of Neuroscience, Western University, 1151 Richmond St. N., London, N6A 3K7 Canada; 3 Queensland Brain Institute, University of Queensland, Brisbane, 4072 Australia; 4 University of Ottawa Brain and Mind Research Institute, 451 Smyth Rd, Ottawa, K1H 8M5 Canada; 5 The Royal’s Institute for Mental Health Research, University of Ottawa, 1145 Carling Ave, Ottawa, K1Z 7K4 Canada; 6 Department of Psychology, Western University, London, N6A 5C2 Canada; 7 School of Psychology, University of Ottawa, 136 Jean-Jacques Lussier, Ottawa, K1N 6N5 Canada

**Keywords:** Circadian rhythms and sleep, Cognitive neuroscience

## Abstract

Sleep resting state network (RSN) functional connectivity (FC) is poorly understood, particularly for rapid eye movement (REM), and in non-sleep deprived subjects. REM and non-REM (NREM) sleep involve competing drives; towards hypersynchronous cortical oscillations in NREM; and towards wake-like desynchronized oscillations in REM. This study employed simultaneous electroencephalography-functional magnetic resonance imaging (EEG-fMRI) to explore whether sleep RSN FC reflects these opposing drives. As hypothesized, this was confirmed for the majority of functional connections modulated by sleep. Further, changes were directional: e.g., positive wake correlations trended towards negative correlations in NREM and back towards positive correlations in REM. Moreover, the majority did not merely reduce magnitude, but actually either reversed and strengthened in the opposite direction, or increased in magnitude during NREM. This finding supports the notion that NREM is best expressed as having *altered*, rather than *reduced* FC. Further, as many of these functional connections comprised “higher-order” RSNs (which have been previously linked to cognition and consciousness), such as the default mode network, this finding is suggestive of possibly concomitant alterations to cognition and consciousness.

## Introduction

Resting state network (RSN) functional connectivity (FC) has been evaluated for a number of compromised and non-wakefulness states, including sedation^1,2^, the vegetative state^3,4^ and sleep^5–8^. These studies suggest that reduced states of conscious awareness are associated with a reduction in the magnitude of RSN FC, particularly for non-sensory, “higher-order” RSNs such as the default mode network (DMN)^9^. Further, higher-order RSNs have been associated with executive cognitive functions such as task shifting^10^ and verbal reasoning^11^. As such, RSN FC configurations can serve as a useful investigative tool for profiling both consciousness and higher-order cognitive activity, albeit in an indirect, inferential manner. Such indirect profiling can be particularly useful for brain states in which direct assessments are theoretically challenging, as in sleep. However, RSN FC in sleep is poorly understood, due to the limited amount of fMRI data during sleep stages such as rapid eye movement (REM) and slow wave sleep (SWS)^12^. These sleep stages are accompanied by dramatic changes to the neurochemical and electrophysiological milieu of the brain. However, it remains to be fully determined how these systems-level changes are reflected in the accompanying changes to RSN FC dynamics, particularly in the non-sleep deprived brain.

REM and non-REM (NREM) sleep are defined by distinct electrophysiological signatures with unique neurophysiological substrates. In particular, REM sleep is dissociated from NREM sleep by the extent of cortical synchrony. NREM sleep is characterized by cortical delta waves, ~ 0.5–2 Hz oscillations that are the consequence of widespread synchronized communication. NREM sleep is typically^13^ further subdivided into NREM1, NREM2, and SWS, during which hypersynchronized cortical oscillatory activity^14^ increases progressively. In contrast, REM is associated with “desynchronized” cortical activity^15^ similar to wakefulness.

Reassuringly, such dramatic changes in EEG signature indeed appear to be reflected in concomitant spontaneous activity FC changes. A range of methodologies have been implemented in prior studies, with each providing unique insight into different aspects of sleep physiology and their respective functions. For example, seed-based correlation analysis has been used to identify a breakdown in the integrity of networks such as the DMN during NREM sleep as compared to wake^8,16^, however this is followed by a recoupling during REM^17^. Region-of-interest (ROI) based correlation approaches have been used to indicate shifts in the organization of RSNs during the transition from wake to NREM sleep^6^, with intra- vs. inter-network comparisons indicating increasing modularity of RSNs during NREM sleep^18^. Despite these findings, independent component analysis (ICA) indicates the general preservation of RSN topology during NREM^19,20^. Taken together, ICA reveals the overall robustness of RSNs during NREM, with seed-based methods revealing more subtle FC modulations within networks. The temporal complexity of DMN and attention network blood oxygen level dependent (BOLD) activity timeseries has also been evaluated as a potential proxy for conscious awareness, indicating a possible reduction during deep sleep^5^. Further, dynamic FC assessments indicate that NREM2 expresses fewer FC transitions than wakefulness^21^, with this reduced capacity to explore FC state space possibly reflecting reduced support for cognition during this stage.

Non-RSN based brain parcellations and graph theoretical results support these findings, indicating global FC reductions during deep sleep, albeit an initial increase in FC during NREM1^22^. Although such parcellations are very useful in generating unbiased, data-driven results, they lack the ready interpretability of RSNs, vis a vis perceptual states and cognition. Moreover, a prior study by our own group^20^ and others^19^ suggests that, despite intra-RSN modulation by sleep, the spatial boundaries of wakefulness RSNs are well preserved across sleep stages, with no new RSNs appearing (despite a directed search for new sleep RSNs^20^). Importantly, these findings of the spatial robustness of the wakefulness RSNs, across all sleep stages, motivates a FC analysis across all sleep stages centered upon wakefulness-based RSN spatial templates. However, to our knowledge, a comprehensive evaluation of inter-RSN FC dynamics across wakefulness and all sleep stages, in healthy non-sleep deprived subjects, has yet to be performed. Accordingly, this complicates our capacity to generate inferences about changes to awareness and cognition during healthy sleep.

A useful prediction of such FC dynamics might be made however, based upon the FC dynamics of the most extensively examined RSN, the DMN; i.e., a progressive deviation away from the wakefulness FC profile during deepening NREM stages^6,8^, followed by a return to wake-like FC during REM^17^. As far as awareness is concerned, the present literature suggests that NREM (particularly SWS), involves both reduced arousal/awareness of the environment, as well as reduced desynchronized neural activity. For example, glucose consumption in NREM is half that of wakefulness^23,24^ and global FC decreases dramatically in SWS^22^. Despite such indications, it is largely the FC of higher-order RSNs specifically, that is modulated by states of cognition and conscious awareness^3,4,9–11^. Consequently, although NREM sleep certainly involves reduced conscious *arousal*, characterized by reduced sensory processing of the environment^25^, it is not entirely clear whether it also manifests a qualitatively *different/altered* state of cognition and conscious awareness. Such a state could manifest correspondingly unique RSN FC patterns, as opposed to merely reduced wake-like FC patterns.

By employing simultaneous EEG-fMRI during sleep, this study had two primary aims: the first aim was to compare inter-RSN FC across all prominent sleep–wake states, in order to determine how changes in RSN FC patterns reflect known electrophysiological differences between NREM and REM. It was hypothesized that inter-RSN FC would trend away from wakefulness-like FC, *in a progressive fashion*, during NREM and subsequently trend back towards wakefulness-like FC in REM. The second aim was to determine whether NREM RSN FC represents merely a reduced version of wakefulness FC, or an altered state of functional connectivity altogether.

## Results

### Edge functional connectivity polynomial fit results

Of the 91 total FC edges, polynomial fits for 49 edges failed to reject the null hypothesis. This suggests that either FC does not change across sleep/wake states for these edges, or that these results were not robust enough to generate conclusions with respect to the alteration of FC across wakefulness and sleep. This is not surprising however, as it would not necessarily be expected that all brain region pairings would change the magnitude or direction of their FC from sleep to wake. Importantly, this also suggests that the remaining edges, which we seek to further understand here, are the most responsive to neurophysiological dynamics across sleep/wake states. Of these remaining 42 edges, six were best described by either linear or cubic fits, with the vast majority, 86% or 36 edges, best described by quadratic fits, in line with our hypothesis. Of these 42 edges, 14 survive Holm-Bonferroni correction, with the vast majority of these (i.e., 12) being quadratic. By contrast, the remaining polynomial patterns represent a relatively small minority of FC edges; only 2 survive Holm-Bonferroni correction. This significant result (Table 1) strongly suggests that whole-brain RSN FC can be best described as deviating away from wakefulness FC during NREM sleep, and returning back towards wakefulness FC in REM sleep.

**Table 1:** Chi square test of the distribution of polynomial fits to resting state network functional connectivity data across wakefulness and sleep.

Linear (N)	Quadratic (N)	Cubic (N)	χ^2^	p
5	36	1	52	< 0.001

The quadratic fit edges were then categorized according to their inflection, with 18 found to be convex, and the remaining 18 being concave. Within each of these quadratic categories however, the majority of edges (13/18 convex and 14/18 concave) moved in a direction towards reversed polarity during NREM (i.e., by either reducing magnitude, or by reversing polarity), and back towards wakefulness polarity during REM. Further, many of these subset edges appear to actually reverse polarity during NREM, instead of merely trending in the direction of reversed polarity. This suggests that FC in NREM sleep is systematically, and specifically, driven in the opposite direction from wake-like connectivity. For example, where two RSNs are positively correlated with one another in wakefulness, by contrast, in NREM sleep FC is driven in the *direction* of negative correlation.

**Figure 1:**
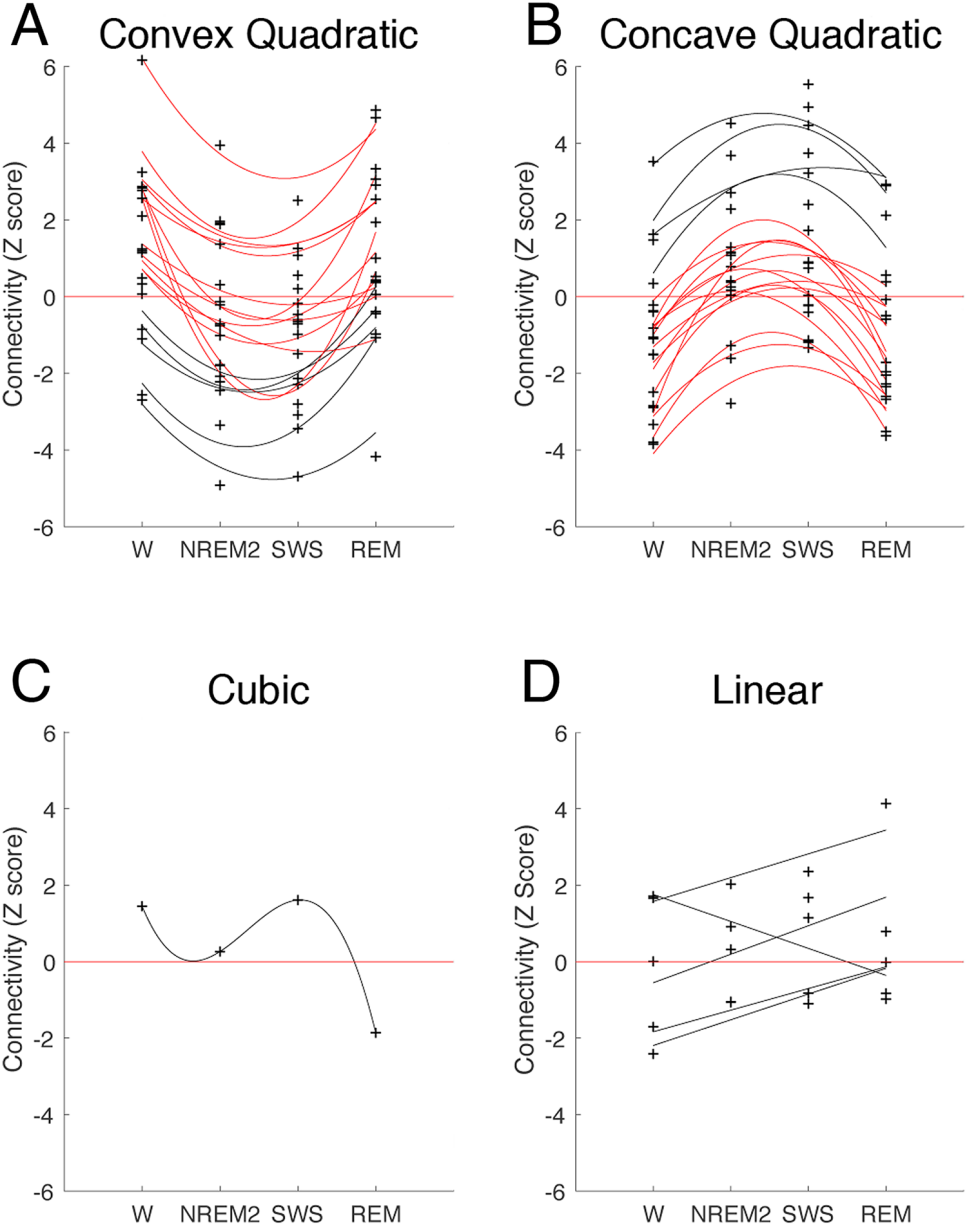
Significant polynomial fits to functional connectivity (FC) data across wakefulness and sleep stages. Plus symbols indicate group-average FC values for a given edge, for a given stage. Units are Fisher r-to-z-transformed full-correlation values, taking into account autocorrelation. FC edges that change in the direction opposite to wakefulness polarity during NREM and return towards wakefulness FC during REM are indicated with red lines. Figures generated using MATLAB (R2019a; mathworks.com). FC edges best described by: (**A**) convex quadratic fits (N = 18; 13 red). (**B**) Concave quadratic fits (N = 18; 14 red). (**C**) Cubic fits (N = 1). (**D**) Linear fits (N = 5). Also see Supplemental Figs. S1 and S2, for FC matrices indicating the direct statistical comparison of edge FC distributions between specific stages. *W* wakefulness, *REM* rapid eye movement, *NREM2* non-REM stage 2, *SWS* slow wave sleep.

The polynomial fits for the significant FC edges are illustrated graphically in Fig. 1, with the subset of convex and concave quadratic edges in which NREM FC moved opposite wakefulness FC indicated in red (also see Supplemental Figs. S1 and S2, for FC matrices indicating the direct statistical comparison of edge FC distributions between specific stages and Supplemental Fig. S3, for histograms of the FC values for each stage). By contrast, the remaining polynomial patterns represent a relatively small minority of FC edges.

### Angular distances between stages

Overall, the above pattern of results, combined with the extant literature, strongly suggests that wakefulness and REM sleep could be characterized as FC states that are most similar to one another, and further, that wakefulness and SWS are most dissimilar (with NREM2 being intermediate between the two). However, despite the fact that the quadratic edges represent the majority of the significant polynomial fit edges, they still only represent a subset of the total number of FC edges (36/91). It is therefore not clear whether it can also be said that overall whole-brain RSN FC changes reflect this pattern. One method for assessing the dissimilarity of sets of features is angular distance. The complete set of features for a given state (in this case, FC edge data) can be assembled into a vector in multidimensional space (with one dimension per FC edge) and angular distances can be calculated between pairs of such vectors, with larger values indicating greater differentiation. We predicted that, overall (i.e., in the comparison of vectors comprising all 91 FC edges), the angular distances amongst the stages would reflect the suggested pattern described above.

**Figure 2:**
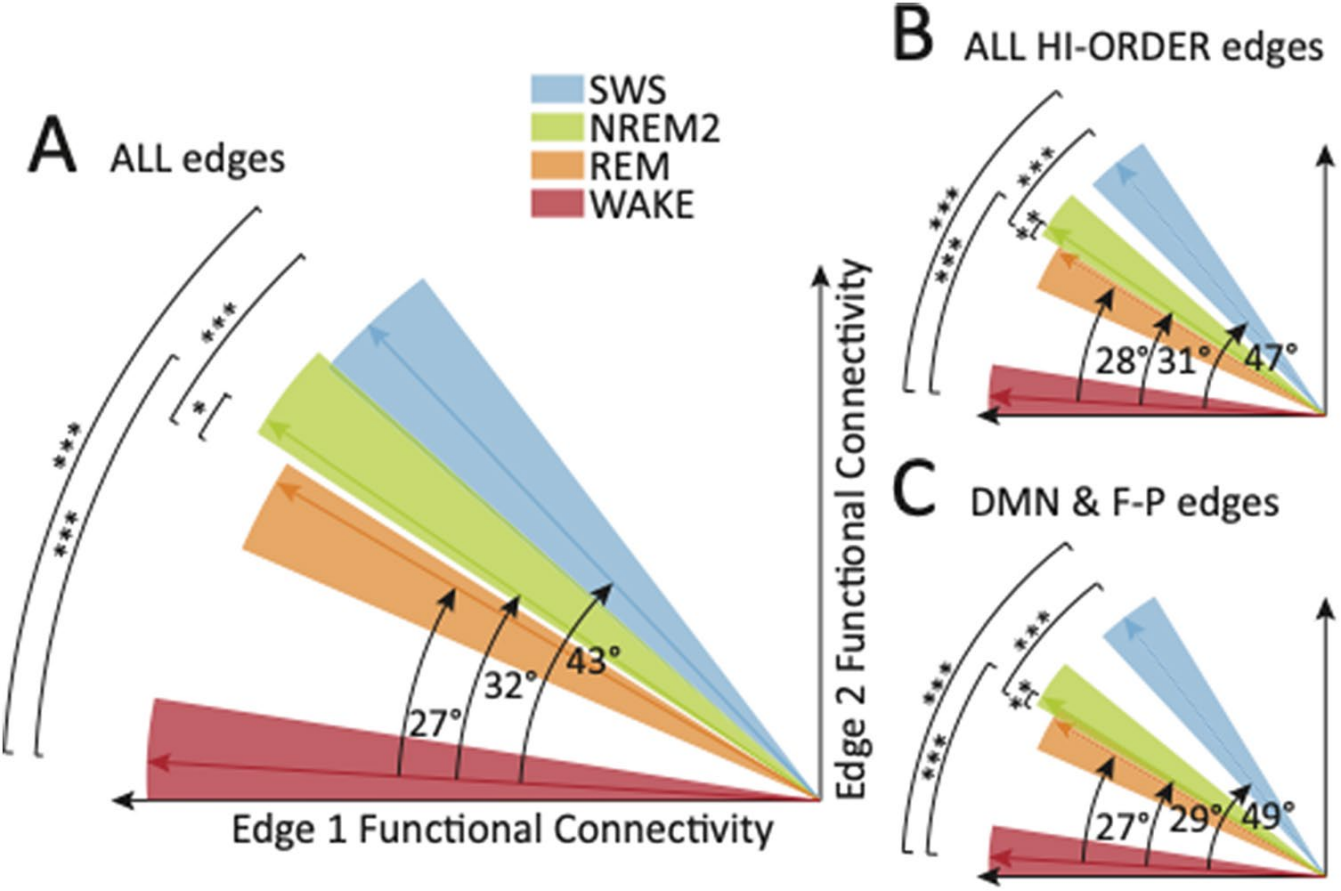
Representative cartoon of the angular distances between vectors representing resting state network (RSN) functional connectivity (FC) in different sleep–wake stages. Vectors exist in multidimensional space, with the number of dimensions dependent on the number of FC edges that are in a given category (e.g., each vector exists in 91-dimensional space for the category “ALL edges”). However, only two dimensions are represented here, for illustrative purposes. Indicated angles are the degree-equivalent of the angular distances between the mean vectors for each stage (indicated as colored arrows), with the mean wakefulness vector always used as the reference point. Angles between sleep stages are not indicated, however, the statistical significance of these differences is indicated by asterisks (single/double/triple asterisks indicate p < 0.05/0.01/0.001, respectively). Note that angles between any pair of vectors actually exist in separate dimensional planes and are only represented in the same plane for illustrative purposes. Also note that angle equivalents between sleep stages are *not* equal to differences in the individual angles relative to wake, as such angles exist in multidimensional space and must be calculated separately. Colored triangles indicate the spread of vectors for each stage, again for illustrative purposes, as they are actually spread across multidimensional space. (**A**) ALL edges (N = 91 dimensions). (**B**) ALL HIGHER-ORDER resting state network edges (N = 70). (**C**) Default mode network (DMN) & Fronto-Parietal network (F-P) edges (N = 46). *REM* rapid eye movement, *NREM2* non-REM stage 2, *SWS* slow wave sleep.

The results (Fig. 2A) confirm that, overall, RSN FC in SWS sleep is indeed driven the furthest away from wakefulness (i.e., the angular distances between the groups of SWS and wake vectors are greatest), whereas RSN FC in REM sleep recovers back to a state that more closely resembles wakefulness (i.e., the angular distances are smallest), with NREM2 being intermediate. Note that only angle equivalents *relative to wakefulness* are presented in Fig. 2. Thus, angle equivalents between sleep stages are not equal to differences in the individual angles relative to wake, since these angles actually exist in multidimensional space, outside of the two-dimensional simplified plane presented in this figure. Importantly, the differences between all NREM vectors (i.e., NREM2 and SWS) and those of both wakefulness and REM were also significant and the differences between the vectors of wakefulness and REM were not significant. The significant differences also survive Bonferroni correction. This indicates that, statistically, REM and wakefulness could not be distinguished from each other on the basis of their overall RSN FC, whereas both NREM2 and SWS can be distinguished from both wakefulness and REM. Based on these results, it is therefore reasonable to describe overall changes in RSN FC in terms of the predicted pattern.

Having found that NREM comprises an altered state (relative to both wakefulness and REM), from the perspective of whole-brain RSN FC, we next asked whether the same could be said from the perspective of the subset of RSNs that have previously been associated with higher-order cognition. This analysis was done in order to better understand the functional significance of the differences in FC between sleep/wake states. Consequently, the angular distance analysis was repeated using the 70 FC edges comprising at least one higher-order RSN node, since the FC of such RSNs has been associated with executive cognitive functions such as goal management^10,26^ and verbal reasoning^11^. The following RSNs were classified as higher-order, on account of their involvement in the manipulation of multimodal information: (anterior and posterior) default mode network (aDMN, pDMN), executive control network (ECN), (left and right) fronto-parietal network (lF-P, rF-P), dorsal attention network (DAN). These RSNs were distinguished from the following “sensory” RSNs, which are primarily involved in the manipulation of unimodal sensory information: Auditory (A), somato-motor (SM), striate-, extrastriate- and ventral stream-visual networks (sV, esV, vsV).

As shown in Fig. 2B, we identified the same pattern in the higher order edges, as in the whole-brain analysis. Indeed, the angular distances between the wake and SWS vectors were significantly different, after Bonferroni correction (angle equivalents were 43° and 47°, respectively, for the whole-brain analysis and the higher-order RSN analysis). While such a result might not be completely unexpected, this is the first study to comprehensively compare higher-order RSN FC across all stages of sleep and wakefulness.

Finally, we asked whether NREM could be distinguished as an altered state, relative to wakefulness and REM, based on the subset of FC edges comprising RSN nodes that have previously been associated with the modulation of conscious awareness, namely, the DMN and F-P RSNs^1,2,9,27,28^. We therefore repeated the angular distance analysis a second time, using vectors comprised of the subset of 46 edges comprising at least one DMN or F-P node. As shown in Fig. 2C, we identified the same pattern as the two prior analyses, with significant angular distance-equivalent angles that were similar to the higher-order RSN analysis (after Bonferroni correction).

### Directional FC changes between wakefulness and NREM

The results of the angular distance analyses suggested that NREM is an altered state of FC, from the perspective of whole-brain RSN FC, and also from the perspective of those RSNs that have previously been associated with executive, higher-order cognition, and with consciousness. However, an angular distance analysis can only tell you that states are different; it doesn’t determine *how* states are different. Consequently, all FC edges were further statistically tested to determine whether the transition from wakefulness to NREM FC manifested; (A) a reduction in the magnitude of wakefulness FC (i.e., negative FC values become less negative, positive values become less positive); (B) an increase in the magnitude of wakefulness FC, or; (C) a reversal of wakefulness FC (i.e., negative FC values become positive, or vice versa).

**Figure 3:**
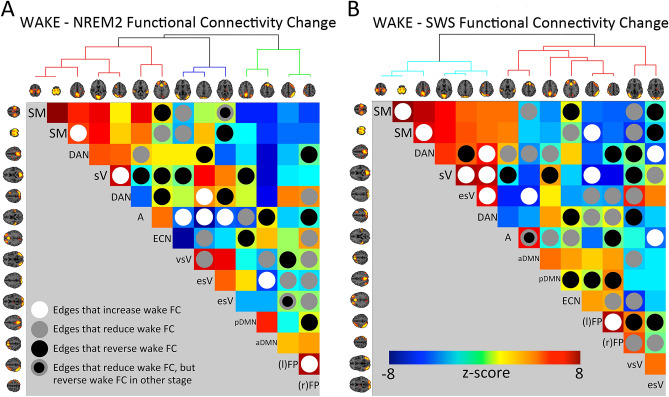
Summary of functional connectivity (FC) edge changes across wakefulness and Non-rapid eye movement (NREM). (**A**) FC changes between wakefulness and NREM stage 2 (NREM2). (**B**) FC changes between wakefulness and slow wave sleep (SWS). Circles indicate FC edges that increase (white), reduce (grey), or reverse (black) the polarity of FC during NREM, relative to wakefulness. FC edges that reduce wakefulness FC during one NREM stage, but reverse wakefulness FC during the other NREM stage are also indicated (grey-black circles). Circles overlaid on top of FC matrices for NREM2 (**A**) and SWS (**B**). Nodes reordered according to hierarchical clustering (hierarchy visualized above matrix). FC matrix colors represent Fisher r-to-z transformed correlations between nodes (taking into account autocorrelation and standard error), with 1-group t-test performed on all participants with data available for a given sleep stage. Select graphical elements of this figure (FC matrices, brain images, cluster hierarchies) were generated using the FSLNets network modeling toolbox (v0.6.3; fsl.fmrib.ox.ac.uk/fsl/fslwiki/FSLNets). *SM* somato-motor, *sV* striate visual, *A* auditory, *DAN* dorsal attention network, *esV* extra-striate visual, *vsV* ventral stream visual, *DMN* default mode network, *ECN* executive control network, *l/rF-P* left/right fronto-parietal.

Remarkably, the results indicate that the majority of significant stage transitions between wakefulness and NREM are either increases, or reversals of FC (see Table 2), rather than reductions of FC (for reference, the specific nodes involved in each of the three stage transitions are indicated in Fig. 3). This finding holds, following Holm-Bonferroni correction (i.e., 6 reversal, 3 increase, and 7 reduction edges). A binomial test indicated that the proportion of increases/reversals (0.62), compared to reductions was higher than predicted (0.5), p = 0.029 (1-sided). Not only was this pattern identified when all FC edges were examined, but, suggestively, it was also identified for the subset of edges comprising higher-order RSN nodes (the binomial test results are marginally significant, however; p = 0.058). It was further identified for the subset of edges comprising DMN or F-P network nodes (the binomial test results are not significant in this case; i.e., p = 0.28). Furthermore, for these same categorizations (whole-brain, higher-order, DMN & F-P), the majority of edges indicate reversals, rather than increases in FC. This last finding reinforces the idea of NREM being an altered state of RSN FC, in contrast to an amplified or reduced state, relative to wakefulness FC.

**Table 2:** Summary of sensory and higher-order resting state network edges involved in reduced, increased or reversed functional connectivity (FC) changes between wakefulness and non-rapid eye movement sleep (NREM).

Edge node types	Significant FC change between wakefulness and NREM		
	Reduction (N)	Increase (N)	Reversal (N)
All edges	21	14	20
Higher-order—higher-order	5	2	5
Higher-order—sensory	11	7	11
Sensory—sensory	5	5	4
DMN or F-P nodes	12	4	10

*DMN* default mode network, *F-P* fronto-parietal network.

Collectively, these results are surprising in that they appear to suggest that, from the perspective of RSN FC, NREM, and SWS in particular, might be a much more active state than previously supposed, given that the majority of FC edges associated with whole-brain connectivity, higher-order cognition, and with consciousness indicated increased or reversed FC, relative to wakefulness. This result is in striking contrast to conventional wisdom that NREM is a state characterized by degraded, reduced or disconnected functional communication between brain regions.

## Discussion

There are four notable results from this study, each with important implications for understanding the RSN FC, and possibly, the associated levels of cognition and awareness, of brain states in which neurophysiology varies dramatically. First, as predicted, between-RSN FC appears to follow the aforementioned neurophysiological trends across wakefulness and sleep (i.e., changing cortical synchrony), for the majority of FC edges that were found to be best described by significant polynomial fits (i.e., 36/42 were quadratic). Consequently, we have identified, for the first time, a potential correspondence between these two distinctive, dynamic processes (i.e., RSN FC and cortical neurophysiology), across the complete spectrum of healthy brain states (i.e., wakefulness, NREM2, SWS and REM).

Second, the direction of this FC change was predictably specific to what the “starting” FC was in wakefulness, for 27/36 of the quadratic fit edges. For example, if edge FC between two RSNs was highly positively correlated in wakefulness, then this FC would trend increasingly in the direction of negative correlation from NREM2 to SWS and then return towards increasing correlation in REM.

Third, the angular distance results indicated that NREM, and SWS in particular, can be considered as an *altered* state (relative to both wakefulness and REM), from three different perspectives; namely, from whole-brain RSN FC; from the subset of higher-order RSN FC, and; from the further subset of DMN and F-P FC. Given previously established associations for the two subsets of RSNs, these findings are, at the very least, consistent with the possibility that NREM manifests both an altered state of higher-order cognition and of conscious awareness, relative to wakefulness. We acknowledge however, that these findings are only suggestive of such an interpretation, and that neither cognition, nor consciousness was measured directly in this study. Nevertheless, these findings could justify future, more direct investigations of this interpretation. It is also worth clarifying that REM can certainly also be viewed as an “altered” state, from a perceptual standpoint. However, while REM is known to manifest bizarre dream content, possibly as a consequence of reduced executive processing^29^, the vivid quality of this content is perhaps more similar, perceptually, to a waking experience, than that of NREM. By contrast, the altered perceptual structure^30^ (e.g., absent narrative content), and radically differentiated NREM FC suggests that the term altered might be more appropriate for NREM, at least relative to wake.

These results support the idea of contrasting functions for NREM and REM, wherein the brain is purposefully driven into a different FC configuration in NREM and is then returned to a wakefulness-like configuration in REM. It is possible that this very specific directional change in RSN FC serves a homeostatic function, such that RSN FC in NREM reduces FC, as it is established during a given day, so that the brain is less biased towards specific RSN connectivity the following day. This could result in cognitive flexibility and thereby facilitate improved adaptability. It is also consistent with the principle of the synaptic homeostasis hypothesis^31^, which asserts that NREM sleep serves to counterbalance accrued wakefulness long term potentiation (LTP) between specific neurons.

The fourth and most important finding was that the majority of RSN FC edges that change significantly between wakefulness and NREM, manifest either an increase in magnitude, or a reversal of the polarity of wakefulness FC during NREM, rather than a mere reduction of FC. As with the angular distance analysis, this result was found to hold from three important perspectives; whole-brain RSN FC, higher-order RSN FC, and the higher-order subset of DMN/F-P FC. With these results, we can further specify the nature in which NREM manifests as an altered state from wakefulness, and thereby flesh out the angular distance results. Namely, NREM seems to manifest as a combination of reversed, increased, and reduced RSN FC, relative to wakefulness, however it appears to be dominated by the first two transition types. Importantly, we believe that the term “altered” best applies to this specific combination of significant RSN FC transitions. We purposely distinguish from the term “reduced”, which we would otherwise use to describe alternative findings in which significant FC transitions were instead dominated by reduced RSN FC.

Moreover, the fact that this finding applies to both the subset of higher-order edges and to the further subset of DMN/F-P edges is, at the very least, consistent with a characterization of NREM as an altered state of higher-order cognition and conscious awareness (more so than was indicated by the angular distance results). Importantly, this finding further contrasts with the characterization of NREM as a state of reduced connectivity, but also highlights the importance of discriminating between three types of RSN FC state transitions, (i.e., reversals, increases and reductions) rather than just increases or decreases in magnitude. Indeed, solely examining magnitude changes in FC during SWS, as in Spoormaker^22^, or even in the present study, leads to the conclusion that NREM predominantly manifests reduced FC, relative to wakefulness. By contrast, the altered character of NREM is only revealed when reversed and increased RSN FC transitions are together contrasted with reduced RSN FC. Notably, amongst the reversal and increasing FC edges, the predominance of reversal edges suggests that at least one of the functions of NREM sleep might be to drive cortical FC as far away from wakefulness FC as possible, further consistent with a homeostatic function.

It is important to note here that the observed reversal of FC is not inconsistent with the findings of other studies that FC is reduced in SWS sleep^22,32^, as FC can trend in the direction of reversed FC without increasing in absolute value. For similar reasons, these findings are also not inconsistent with indications that energy consumption is reduced in NREM^33,34^.

Limitations of this study include the low number of participants with SWS (N = 9) and REM (N = 6). This limitation directly impacts any comparison made with these stages, particularly wake-REM comparisons, and including the polynomial fitting. On a related note, only four participants experienced what could be described as a complete first sleep cycle, with all four stages represented (i.e., also including NREM1). Thus, the majority of participants may be better described as having experienced a nap, rather than a night of sleep. However, as far as we are aware, this comprises the largest amount of healthy non-sleep deprived EEG-fMRI sleep data collected for these stages within a single study, given the challenges in sustaining sleep in the MRI scanner environment. Nevertheless, the aforementioned findings must be interpreted with caution, while taking these limitations into account.

A further limitation concerns the suggested implications with respect to conscious awareness and cognition during sleep. As mentioned above, measures of these qualities during sleep were not directly acquired. Nonetheless, we believe that this particular limitation is more reflective of the practical limitations of assessing consciousness during sleep more generally, given the relative isolation of the brain from the external environment. The most notable alternative solution to this limitation, i.e., reports derived from awakenings^35^, are very useful, however they are burdened with their own limitations. For example, it is unclear whether the awakening process interferes with memory encoding. It therefore remains possible that states of internal mentation and even consciousness were present prior to the awakening, with the report representing a false negative. Indeed, such memory encoding failures are suggested by intraoperative awareness^36^. We therefore consider the presentation of these findings to be an important step in addressing this practical challenge. Given the wealth of knowledge about the functional significance of RSNs, their interrogation across sleep/wake states has the potential to be used as a proxy for assessing capacities such as consciousness, which is otherwise challenging to determine during sleep.

We also acknowledge that the present study only addresses inter-RSN FC, not intra-RSN FC. This was a conscious limitation in order to focus on the relevance of RSNs to higher order cognition. Further, the external dataset (comprising independent components specific to a 20 model-order ICA decomposition) used to derive the RSNs in this study, was also selected with this focus in mind, even though different model-order ICA decompositions might yield RSNs with better representation in subcortical regions, for example. We would also add that in our previous study, utilizing the same dataset^20^, we found RSNs to be largely preserved across vigilance states, when using an ICA approach. We acknowledge that within-network FC is modulated by sleep, as evidenced by seed-based analysis for example^8^, however the overall spatial integrity of an RSN is largely preserved, with no new RSNs appearing in any sleep stages, according to our prior study.

Finally, we note that preprocessing is known to shift FC distributions, however there is as yet no consensus on the ideal set of preprocessing steps for resting state data^37^. Although we believe that any distribution shifts associated with our preprocessing steps are appropriate (being the consequence of the removal of non-neuronal noise artifacts), FC categorizations, in particular FC reversals, should be interpreted within the context of the preprocessing used in this analysis. Within this context however, FC histograms are reassuringly similar, suggesting that FC comparisons across states are based upon similar reference points. We further acknowledge that other preprocessing steps could lead to different, albeit complementary insights.

Future studies should follow up on one of the key findings of this study, that many RSN FC edges appear to reverse FC during NREM. A longitudinal study would be ideal for determining whether the strength of this reversal corresponds to the FC strength of a given edge on a given day. If so, this would provide further support for the idea that NREM serves a homeostatic function, at the level of RSN FC. It would also be worth investigating edges that don’t appear to change as a function of sleep–wake state, in addition to intra-RSN FC. In this study, 54% of edges were, statistically, best described by a horizontal line across wakefulness and sleep, suggesting either that they genuinely do not change, or that our study did not obtain sufficient data to identify a change. Although given the number of positive results, we feel the likelihood of the former explanation is unlikely. It is also important to investigate other functional roles for this FC reversal in SWS; it is already known that NREM plays a key role in memory consolidation and relates to inter-individual differences in human intelligence^38,39^, for example. One means of investigating such functional roles might be to leverage EEG by identifying connections between RSN FC in different sleep stages and EEG-defined frequency band power dynamics, or phasic events such as sleep spindles and K complexes (all with known functional associations).

In summary, this study demonstrated for the first time that inter-RSN FC appears to be modulated in accordance with changes in neurophysiology across wakefulness and all sleep stages, including REM. It further suggested that NREM, and SWS in particular, progressively modulates RSN FC in a directional fashion, opposite to that of wakefulness, thereby implying a possible wakefulness/NREM homeostatic function. To our surprise, this directional change went as far as reversing FC and strengthening it in the opposite direction. When these changes in FC were tested more explicitly between wakefulness and NREM, across all edges, it was revealed that the majority of whole-brain RSN FC changes involve either *increases or reversals*, rather than mere *reductions*, in the strength of FC. This finding also applied to the subset of higher-order RSN edges and a further subset of DMN & F-P edges, which is suggestive of the possibility that NREM might manifest altered, rather than simply reduced, higher-order cognition and conscious awareness.

## Materials and methods

### Participants

Thirty-six healthy right-handed adults (21 female) 18–34 years of age (M = 23.7, SD = 3.6), were recruited for this study. Of the 36 participants who met the study inclusion criteria, data for 34 participants (21 female, M = 23.7, SD = 3.7) was included in the analysis (one participant withdrew from the study due to discomfort; another did not sleep during the EEG-fMRI session, but did have wake resting state data, however this data was excluded). An a priori statistical power analysis was not performed (there are currently no established procedures for performing a power analysis for resting state network fMRI studies, as there are for task-based fMRI studies), however the number of subjects included is consistent with previous studies investigating RSNs in sleep^5,7,8^. All participants were non-shift workers and medication-free, with no history of head injury or seizures, and had a normal body mass index (< 25). Participants were required and reported to be non-smokers, who drink no more than 2 caffeinated drinks per day, and no more than 14 alcoholic drinks in a week. On the day of the EEG-fMRI recording, participants were not allowed to consume caffeinated, alcoholic, or nicotine products. Further, all scored < 10 on the Beck Depression^40^ and the Beck Anxiety^41^ Inventories and had no history or signs of sleep disorders, as indicated by the Sleep Disorders Questionnaire^42^. All participants were required to keep a regular sleep–wake cycle (bed-time between 22h 00 and 24h 00, wake-time between 07h 00 and 09h 00) and to abstain from taking daytime naps at least 7 days prior to, and throughout participation in the study. Compliance with this schedule was monitored using both sleep diaries and wrist actigraphy (Actiwatch 2, Philips Respironics, Andover, MA, USA). All participants met the MRI safety screening criteria. In addition, participants were given a letter of information, provided informed written consent before participation, and were financially compensated for their participation. This research was approved by the Western University Health Science Research Ethics Board. All the procedures were carried out in accordance with relevant guidelines.

### Experimental design

Each participant underwent a screening/orientation session one week prior to the experimental sleep session. The experimental sleep session took place between 21h 00 and 24h 00, during which time simultaneous EEG-fMRI was recorded while participants slept in the scanner. It consisted of a 5-min structural scan, followed by an eyes-closed wake resting state scan. Participants were then informed that they were free to fall asleep in the scanner. Lights out was at the participant's normal bedtime (~ 22h 00). This period lasted for approximately 2 h. Following the sleep session, participants were allowed to sleep in the nearby sleep laboratory for the remainder of the night.

### Polysomnographic recording and processing

#### EEG recording parameters

EEG was recorded using a 64-channel magnetic resonance (MR)-compatible EEG cap (Braincap MR, Easycap, Herrsching, Germany) and two MR-compatible 32-channel amplifiers (Brainamp MR plus, Brain Products GmbH, Gilching, Germany). EEG caps included scalp electrodes referenced to FCz. Two bipolar electrocardiogram (ECG) recordings were taken from V2-V5 and V3-V6 using an MR-compatible 16-channel bipolar amplifier (Brainamp ExG MR, Brain Products GmbH, Gilching, Germany), synchronized to the MRI scanner acquisition using a Brain Products “SyncBox” (Brain Products GmbH, Gilching, Germany). These signals were acquired in addition to the drop-down ECG lead in the EEG cap, in order to acquire high-quality ECG to better visualize the QRS complex used in the subsequent ballistocardiographic (BCG) artifact correction procedure, described below. Using high-chloride abrasive electrode paste (Abralyt 2000 HiCL; Easycap, Herrsching, Germany), electrode–skin impedance was reduced to < 5 KOhm. In order to reduce movement-related EEG artifacts, participants' heads were immobilized in the MRI head-coil using foam cushions. In addition, the position of the participant was adjusted such that they were 40 mm offset from iso-centre. This procedure has been found to reduce BCG artifacts by as much as 40%^43^ making BCG artifact correction more straightforward. EEG was digitized at 5000 samples per second with a 500-nV resolution. Data were analog filtered by a band-limited low pass filter at 500 Hz and a high pass filter with a 10-s time constant corresponding to a high pass frequency of 0.0159 Hz. Data was transferred via fiber optic cable to a personal computer and recorded using Brain Vision Recorder Software, Version 1.x (Brain Vision, Gilching, Germany).

#### EEG data processing

**Figure 4:**
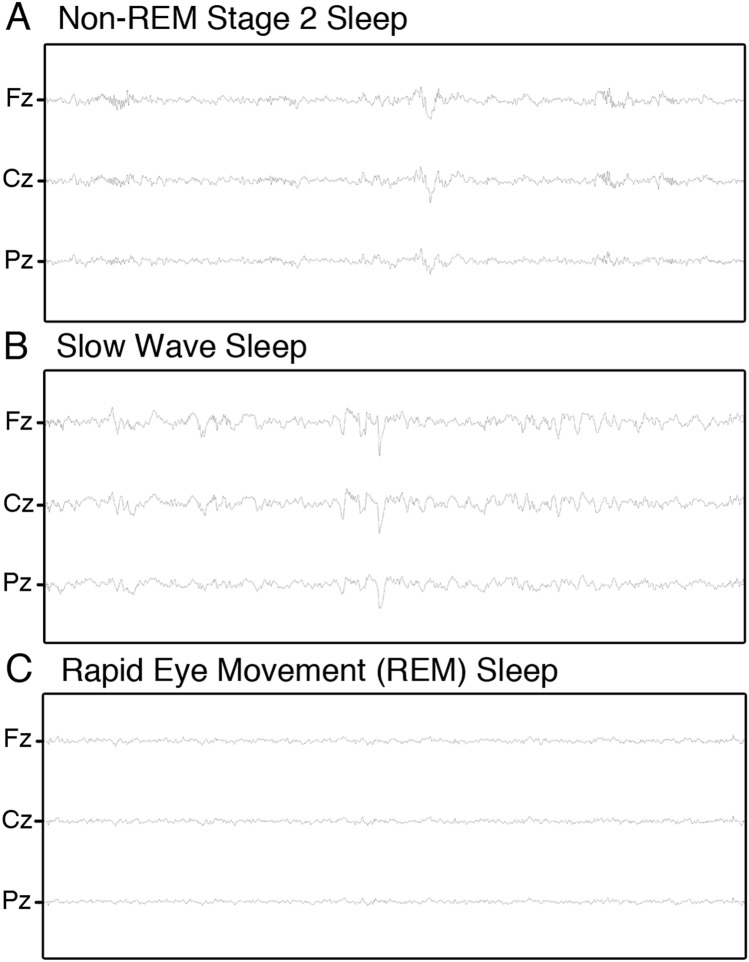
Screenshots of residual gradient- and BCG artifact-corrected EEG tracings for each sleep stage used in the analyses. (**A**) Non-REM stage 2 (**B**) Slow wave sleep (**C**) Rapid eye movement (REM) sleep.

EEG scanner artifacts were removed in two separate steps. First, MRI gradient artifacts were removed using an adaptive average template subtraction method^44^ implemented in Brain Products Analyzer, and down-sampled to 250 Hz. In the second step, the R-peaks in the ECG were semi-automatically detected, visually verified, and manually adjusted when necessary, to correct both false positives and false negative r-peak detections. Then, adaptive template subtraction^45^ was used to remove BCG artifacts time-locked to the R-peak of the QRS complex of the cardiac rhythm. After these two steps, the quality of the data was visually verified and the amplitude of the residual artifacts time-locked to the r-peaks was inspected. An independent component analyses (ICA) based approach^46,47^ was applied to remove any remaining BCG residual artifact if the peak of the maximum amplitude of the residual artifact exceeded 3 μV during the QRS complex (e.g., 0 to 600 ms). Finally, a low-pass filter (60 Hz) was applied to the EEG data, which were then re-referenced to averaged mastoids. Samples of EEG traces after correction are shown, for each sleep stage (i.e., NREM2, SWS and REM), in Fig. 4. Following the artifact correction, sleep stages were manually scored by an expert in accordance with standard criteria^13^ using the “VisEd Marks” toolbox (https://github.com/jadesjardins/vised_marks) for eeglab.

### MRI imaging acquisition and processing.

#### Recording parameters

Brain images were acquired using a 3.0 T TIM TRIO magnetic resonance imaging system (Siemens, Erlangen, Germany) and a 64-channel head coil. A structural T1-weighted MRI image was acquired for all participants using a 3D MPRAGE sequence (TR = 2300 ms, TE = 2.98 ms, TI = 900 ms, FA = 9°, 176 slices, FoV = 256 × 256 mm^2^, matrix size = 256 × 256 × 176, voxel size = 1 × 1 × 1 mm^3^). Multislice T2*-weighted fMRI images were acquired during the sleep session with a gradient echo-planar sequence using axial slice orientation (TR = 2160 ms, TE = 30 ms, FA = 90°, 40 transverse slices, 3 mm slice thickness, 10% inter-slice gap, FoV = 220 × 220 mm^2^, matrix size = 64 × 64 × 40, voxel size = 3.44 × 3.44 × 3 mm^3^). In order to obtain EEG with time-stable artifacts, which aligned to the timing of the EEG recordings, the MR scan repetition time was set to 2160 ms, such that it matched a common multiple of the EEG sample time (0.2 ms), the product of the scanner clock precision (0.1 μs) and the number of slices (40) used^48^.

#### Functional data classification

To be included in the fMRI analysis, the EEG had to be visibly movement artifact-free. Volumes were classified as wake, NREM1, NREM2, SWS or REM in 20 s epochs. Wake data used in the analysis was taken from the wake resting state session only, despite wake segments being present in the sleep session data. This was to avoid including wake periods contaminated with variable levels of drowsiness/sleep inertia from preceding sleep episodes of varying sleep depth. Following sleep scoring, a single epoch of fMRI data was extracted from the total set of functional volumes, for each participant who had data available for a given stage.

The inclusion criteria for fMRI epochs used in the final analysis was done in such a way as to maximize the use of the available data, while also equalizing epoch lengths between stages and across participants. The length of the epoch extracted per participant was determined by considering the minimum length time series available amongst all the participant data for a given stage. For example, if the smallest epoch available for a given participant was 4 min, then a single 4-min NREM2 epoch was extracted from the data available for all participants with NREM2 data.

#### Wake data acquired/extracted for analysis

34 participants included in the analysis had at least 150 volumes (approximately 5^1^/_2_ min) worth of data. One participant had data recorded with different acquisition parameters, so their wake data was excluded, leaving a total of 33 participants, each with 150-volume epochs used in the final analysis. For the direct comparison of stages, the 150 volumes were truncated (see below) to match the number of volumes available in a given sleep stage.

#### Sleep stage data acquired/extracted for analysis

Overall, participants managed to obtain the full spectrum of sleep stages (NREM1, NREM2, SWS and REM sleep). Given the significant challenges of sustaining sleep in an MRI scanner environment (due to noise and participant comfort), on an individual basis the majority of participants maintained sleep in only a subset of the sleep stages of interest, for a duration long enough to be considered sufficient for FC analysis. Despite these challenges, 4 participants did manage to transition through all three sleep-stages of interest (NREM2, SWS and REM), with a sufficient duration of sleep in a given stage for the FC analysis (see Supplemental Table S1 for sleep macrostructural data for each participant with some amount of usable sleep data; note that “Wake” in this table refers to awakenings during sleep, not separately acquired wake resting state data).

Sleep stage NREM1 was mostly unavailable, however considering the brief and transitional nature of this stage, it was justifiably eliminated from the analysis at the expense of exploring interesting FC changes that might occur during the sleep onset process; which would likely require an experimental approach tailored to study sleep onset per se. The majority of participants (24 out of 33) had enough NREM2 sleep data to match the available wake data (150-volume epochs). In the case of SWS, 9 participants had at least 133-volumes (4.8 min) of data. Six participants had at least 129-volumes (4.6 min) of REM data. For the direct comparison of sleep stages, NREM2 and SWS data was truncated (see below for details).

#### Functional data truncation

In order to have equal length epochs to satisfy the requirements of the analysis approach used in the direct inter-stage comparisons (see Supplemental Figs. S1 and S2), data for the stage with more volumes available per subject was truncated to the length of the stage with fewer volumes available (see Table 3 for a summary of the number of volumes and subjects used in different stage comparisons). For the wake *vs*. SWS comparison, wake epochs were truncated to the length of the shorter SWS epochs (i.e., 133 volumes). Similarly, in the comparison of wake vs. REM, wake epochs were truncated to the length of the shorter REM epochs (i.e., 129 volumes). Likewise, NREM2 epochs were reduced to 133 volumes in the NREM2 vs. SWS comparison. Notably, this reduction allowed for two datasets to be re-included in the analysis, resulting in 26 133-volume epochs for the NREM2 vs. SWS comparison. Similarly, 26 129-volume NREM2 epochs were used in the NREM2 vs. REM comparison. Finally, SWS epochs were truncated to 129 volumes for the SWS vs. REM analysis.

**Table 3:** Sample sizes and number of functional magnetic resonance imaging (fMRI) volumes used in comparisons of functional connectivity (FC) between stages.

Comparison	N/number of fMRI volumes			
	Wake	NREM2	SWS	REM
Primary analysis	33/150	24/150	9/133	6/129
**Between-stage FC comparisons presented in Supplemental** **Figs.** **S1****and** **S2**				
Wake vs. NREM2	33/150	24/150		
Wake vs. SWS	33/133		9/133	
Wake vs. REM	33/129			6/129
NREM2 vs. SWS		26/133	9/133	
NREM2 vs. REM		26/129		6/129
SWS vs. REM			9/129	6/129

*REM* rapid eye movement, *NREM2* non-REM stage 2, *SWS* slow wave sleep.

#### Preprocessing

Each sleep and wake epoch was individually preprocessed using the Oxford Centre for Functional Magnetic Resonance Imaging of the Brain Software Library (FMRIB, Oxford U.K.; FSL version 5.09^49^). Functional volumes within each epoch were realigned using FSL's MCFLIRT tool^50^ which performs rigid body transformations. Non-brain voxels were also extracted using FSL's BET tool^51^. Volumes were spatially smoothed using a Gaussian kernel of 5 mm full-width at half-maximum (FWHM) and high-pass temporal filtered (Gaussian-weighted least-squares straight line fitting, FWHM = 2000 s). Functional volumes were then coregistered to the MNI152 standard space (McConnell Brain Imaging Centre, Montreal Neurological Institute) using 12 degree-of-freedom affine registration. Finally, each epoch was individually cleaned of non-neuronal artifacts using the FIX plug-in for the FSL package^52,53^, an automatic noise detection and removal algorithm. Prior to using FIX, FSL's MELODIC tool^54^ was used to generate ICs for each epoch. MELODIC's default dimensionality estimation function automatically estimates the number of ICs by performing a Bayesian analysis. FIX then assessed each of these ICs as noise or signal, after generating more than 180 distinct spatial and temporal features of each IC and feeding these into a multi-level classifier. ICs classified as noise were then subtracted from the ICA mixing matrix and a new set of functional volumes was generated.

### Functional connectivity analysis

The FC analysis was carried out in a number of stages. First, pseudo times series were generated for each RSN. These were created by first spatially regressing 20 independent component (IC) templates (derived from a separate healthy waking RSN ICA study^55^) onto the single-subject 4D epochs available for each sleep stage, using FSL's dual_regression function^56^. External IC templates were used to avoid circular analysis^57^. The regression of waking spatial templates onto sleep stage data was justified by our prior study^20^, in which we identified the persistence of waking RSNs during each stage of sleep using ICA (despite known modulation of within-RSN FC by sleep, as identified by other methods, such as seed-based correlation analysis^8^). The spatial regression produced a set of 20 beta values (i.e., one beta value per IC) for each volume of functional data, reflecting how well each of the 20 ICs were represented at each time point. Each IC therefore had a series of beta values across all time points, which was treated as a pseudo time series, for further FC analysis. These pseudo-time series were used as inputs for the FSLNets network modeling toolbox (v0.6.3; http://fsl.fmrib.ox.ac.uk/fsl/fslwiki/FSLNets). Notably, the 20 external IC templates included non-neuronal ICs. FSL image viewer FSLEyes and the FSLNets' ts_spectra function were used to respectively assess the spatial configuration and power–frequency spectra of each of the 20 ICs, so that noise-related ICs might be excluded from the FC analysis. The time courses of six ICs that were assessed as noise-related were regressed out of all other time series and then deleted, leaving 14 RSN time series. Next, full-correlation matrices were generated from these 14 RSN time series, at the single subject level, using FSLNets’ nets_netmats function, resulting in 91 unique FC edges for each sleep stage, for each participant (i.e., there are 91 unique pairs amongst 14 RSNs). Full correlation values were converted to z-scores using the Fisher r-to-z transform, with corrections made for degrees of freedom, taking into account autocorrelation.

### Polynomial fitting to edge functional connectivity data across wakefulness and sleep

In order to test our main hypotheses, we assessed the pattern of FC changes across all sleep stages for a given FC edge. First, FC data was stage-coded, such that for any given FC edge; the individual wake FC values comprised y-axis values, and each of these was assigned a corresponding x-axis value of “1”; the NREM2 FC values were assigned an x-axis a value of “2”; the SWS FC values were assigned an x-axis value of “3”, and; the REM values were assigned an x-axis value of “4”. Next, first-, second- and third-order polynomials (i.e., linear, quadratic and cubic curves) were each fit to a scatterplot of this data, for each edge. The quality of fit for each curve was assessed by calculating a coefficient of determination (i.e., an R-square value). Next, permutation hypothesis testing was carried out to test for the statistical likelihood of the individual R-square values for each curve fit. Each value was compared with sample distributions of R-square values, generated by resampling the data (i.e., a null distribution of R-square values was generated by randomly assigning FC data for a given edge to different sleep stages, then calculating new R square values for each polynomial type, and iterating this procedure 10,000 times).

**Figure 5:**
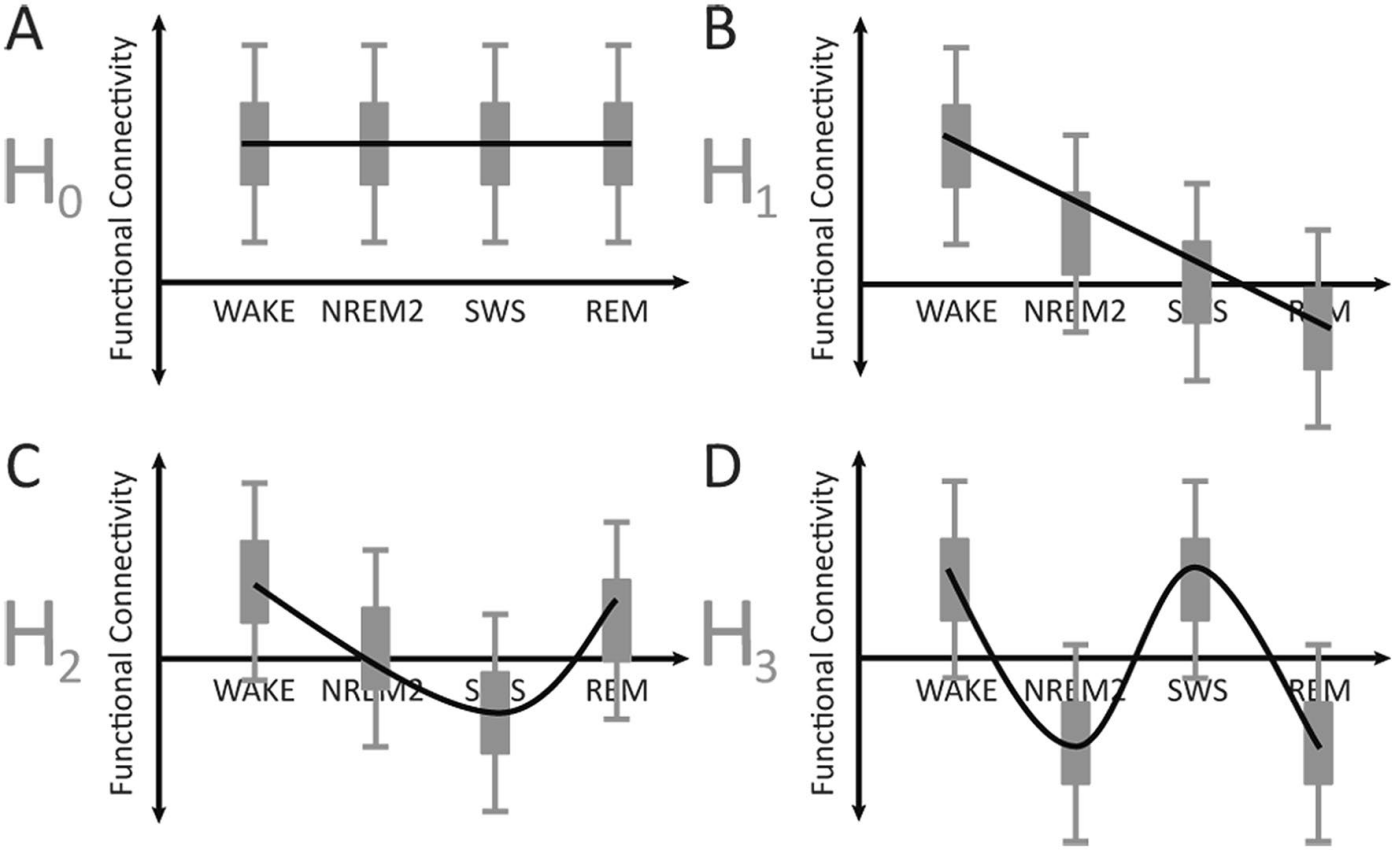
Cartoon of possible polynomial fits for functional connectivity (FC) data across wakefulness and sleep. (**A**) Null hypothesis (H_0_); first-order polynomial, horizontal line fit. (**B**) Alternative hypothesis 1 (H_1_); first-order polynomial, non-horizontal line fit. (**C**) Alternative hypothesis 2 (H_2_); second-order polynomial, quadratic line fit. (**D**) Alternative hypothesis 3 (H_3_); third-order polynomial, cubic line fit. *REM* rapid eye movement, *NREM2* non-REM stage 2, *SWS* slow wave sleep.

Next, each of the 91 edges was assessed as being best described by one of the three polynomial functions, i.e., a first-order non-horizontal line, a second-order quadratic function, or a third-order cubic function (see Fig. 5 for a cartoon of possible fits corresponding to the null and alternate hypotheses). The selection criteria for the best fit was as follows: (1) if only one type of polynomial fit was significantly different from a horizontal line for a given edge, then the pattern of FC change for that edge was categorized as being best described by that fit, (2) if multiple fits were statistically significant, the polynomial fit with the highest R-square value was used to best describe the pattern of FC change for that edge, and (3) if no statistically significant best-fits were identified, then that edge was categorized as being best described by a flat, horizontal line (i.e., no significant changes in edge FC across the sleep stages, in accordance with the null hypothesis; see top left panel of Fig. 5).

Once all the edges were categorized according to best polynomial fit, a one-variable chi-square test was performed to examine the distribution of polynomial fits that differed significantly from a horizontal line.

### Angular distances between stages

Angular distances were evaluated in order to determine the dissimilarity between each of the sleep stages and wakefulness. This was done by first assembling relevant edge FC values for a given participant in a given stage into a single vector. For example*,* for NREM2 there were 24 vectors in total, corresponding to the 24 participants with useful NREM2 data. For comparisons of all FC edge data, each of these 24 NREM2 vectors was comprised of 91 values, corresponding to the 91 unique FC edges amongst the 14 RSNs assessed in this study. By contrast, for comparisons of the subset of DMN & F-P edges, each of the 24 NREM2 vectors was comprised of 46 values, corresponding to the 46 FC edges that comprised either DMN or F-P nodes. Next, angular distances were calculated between mean vectors for each stage (i.e., vectors comprised of edge FC values that have been averaged across participants for a given stage). Angular distances were calculated between pairs of vectors according to the following formula:

1 $${\text{Angular}} \;{\text{distance}} = \frac{{\cos^{ - 1} \left( {\frac{A \cdot B}{{\parallel A\parallel \parallel B\parallel }}} \right)}}{\pi }$$

where A, B are the vectors of interest, $A \cdot B$ is the vector dot product, and $\parallel A\parallel \parallel B\parallel$ are the vector lengths.

In order to evaluate the statistical significance of the differences between the vectors belonging to each stage, a non-parametric MANOVA was performed^58,59^. In this case, Eq. (1) was used to evaluate angular distances between vectors defined by single-subject edge FC data for each stage. The test statistic is a multivariate analogue of the F-ratio, as follows:

2 $$F = \frac{{SS_{A} /\left( {a - 1} \right)}}{{SS_{W} /\left( {N - a} \right)}}$$

where the numerator is the between-groups variance and the denominator is the within-groups variance. SS_A_ is the between-group angular distance sum of squares (SS), calculated as total SS (SS_T_) − SS_W_, SS_W_ is the within-group angular distance sum of squares. SS is calculated as the angular distances amongst all single-subject vector pair combinations, divided by the relevant number of vectors, as per^58,59^, a = number of groups, N = number of vectors.

A null distribution of this statistic was created by resampling the data (i.e., by randomly assigning the vectors to different sleep stages, calculating new pseudo F-statistics and iterating this procedure 10,000 times). A P-value was then calculated for the actual F value by comparing it to the permuted sample distribution. A significant F value was followed by a posteriori testing, in which t-statistics for specific pairs of sleep–wake stages were calculated as the square root of the F-statistic above, as per^58^, with statistical significance calculated using the same resampling technique.

Finally, this set of procedures was repeated for vectors comprised of the subset of FC edges comprising higher-order RSN nodes (i.e., 70 edges), and, for the further subset of FC edges comprising DMN or F-P nodes (i.e., 46 edges).

### Directional FC changes between wakefulness and NREM

Next, in order to test our second aim (i.e., to determine whether NREM FC is better described as a reduced version of wakefulness functional connectivity, or as an alternate state), all edge FC changes were tested to determine whether NREM sleep manifested; (A) a reduction in the magnitude of wakefulness FC (i.e., negative FC values become less negative, positive values become less positive); (B) an increase in the magnitude of wakefulness FC, or; (C) a reversal of wakefulness FC (e.g., negative FC values become positive and vice versa). A and B were accomplished by first calculating, for each edge, a t-statistic based on the actual wake and NREM FC values (i.e., between wake and NREM2 FC values, and, separately, between wake and SWS FC values), and then comparing this t-statistic value against a null distribution of t-statistic values, generated by the same permutation hypothesis testing method described in the polynomial-fitting analysis, above. C was accomplished by first calculating, for each FC edge, a 1-sample t-statistic for the wake FC values, and separate 1-sample t-statistic values for each of the NREM FC data (i.e., for NREM2 and SWS). These t-statistics were compared to null distributions generated using permutation hypothesis testing (e.g., if wake was being compared with NREM2, then the category labels for these two stages were permuted). If, for a given edge, the t-statistic was significant in one direction for wake, and further the t-statistic was significant in the opposite direction, for either of the NREM stages, then that edge was categorized as having reversed its FC across wakefulness and NREM. Finally, binomial tests were performed on the distribution of these types of change (i.e., increases and reversals vs. reductions).

## Supplementary Information

Supplementary Information.
