# Supplementary material for: Reversed and increased functional connectivity in non-REM sleep suggests an altered rather than reduced state of consciousness relative to wake
Source: Sci Rep. 2021 Jun 7;11:11943. doi: 10.1038/s41598-021-91211-5 (PMC8184935; doi:10.1038/s41598-021-91211-5)
Supplement: Supplementary file 1 — Supplementary Information. [file 41598_2021_91211_MOESM1_ESM.pdf]

Title: Reversed and increased functional connectivity in non-REM sleep suggests an altered rather than reduced state of consciousness relative to wake

Houldin, Evan[a,b,c], Fang, Zhuo[a,d], Ray, Laura B.[a,e], Stojanoski, Bobby[a], Owen, Adrian M.[a,f], Fogel, Stuart M.[a,d,e,f,g,1]

[a]Brain & Mind Institute, Western Interdisciplinary Research Building, Western University, London, Canada, N6A 5B7, [b]Department of Neuroscience, Western University, 1151 Richmond St. N., London, Canada, N6A 3K7, [c]Queensland Brain Institute, University of Queensland, Brisbane, Australia, 4072, [d]University of Ottawa Brain and Mind Research Institute, 451 Smyth Rd, Ottawa, Canada, K1H 8M5, [e]The Royal's Institute for Mental Health Research, University of Ottawa, 1145 Carling Ave, Ottawa, Canada, K1Z 7K4, [f]Department of Psychology, Western University, London, Canada, N6A 5C2, [g]School of Psychology, University of Ottawa, 136 Jean-Jacques Lussier, Ottawa, Canada, K1N 6N5,

[1]Corresponding Author:

Dr. Stuart Fogel
Associate Professor,
School of Psychology
Director, Sleep Neuroscience
University of Ottawa Institute for Mental Health Research,
University of Ottawa
Ottawa, Ontario, Canada
(613) 562-5800 x4295
sfogel@uottawa.ca
socialsciences.uottawa.ca/sleep-lab/

SUPPLEMENTAL FIGURES

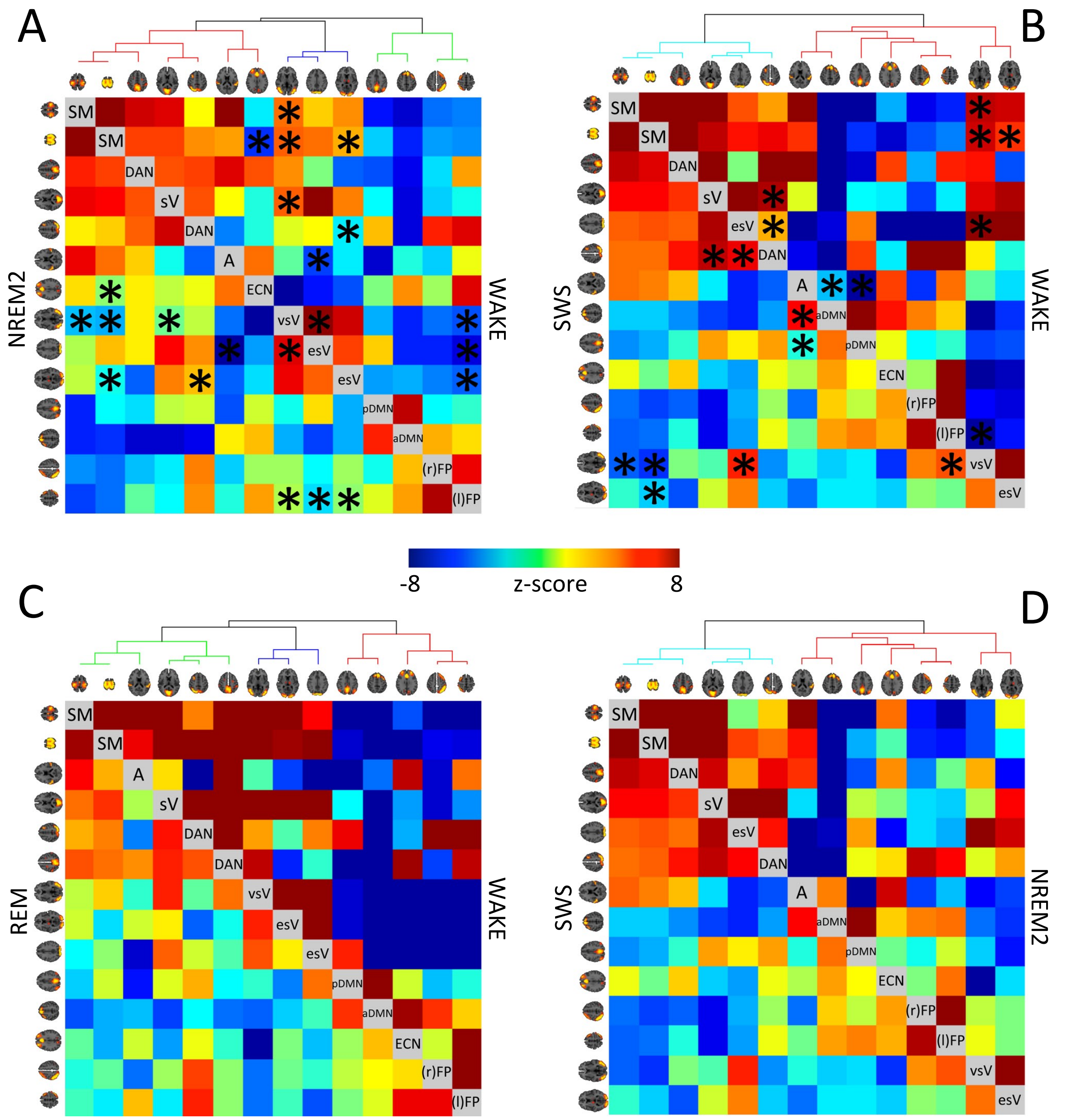

Figure S1. Full-correlation functional connectivity (FC) matrices for 14 resting state networks (RSNs) across wakefulness and sleep. Nodes reordered according to hierarchical clustering of the below-diagonal sleep stage (hierarchy visualized above each matrix). Colors represent Fisher r-to-z transformed correlations between nodes (taking into account autocorrelation and standard error), with 1-group t-test performed on all subjects with data available for a given sleep stage. Asterisks indicate significant differences ($p < 0.05$ minimum) in edge FC between stages, as determined by univariate 2-sample t-tests. Select graphical elements of this figure (FC matrices, brain images, cluster hierarchies) were generated using the FSLNets network modeling toolbox (v0.6.3; fsl.fmrib.ox.ac.uk/fsl/fslwiki/FSLNets). (A) non-rapid eye movement stage 2 (NREM2) matrix below, wake above diagonal. (B) slow wave sleep (SWS) below, wake above diagonal. (C) REM below, wake above diagonal. (D) SWS below, NREM2 above diagonal. SM=somato-motor, sV= striate visual, A=auditory, DAN=dorsal attention network, esV=extra-striate visual, vsV=ventral stream visual, DMN=default mode network, ECN=executive control network, l/rF-P=left/right fronto-parietal.

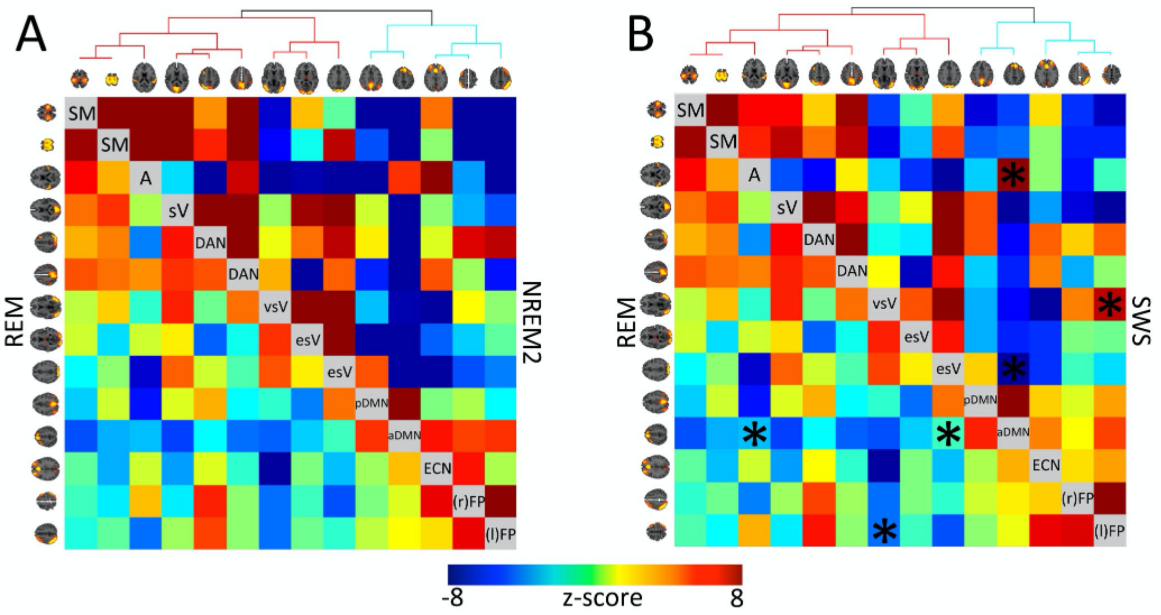

Figure S2. Full-correlation functional connectivity (FC) matrices for 14 resting state networks (RSNs). Nodes reordered according to hierarchical clustering of the below-diagonal sleep stage data (hierarchy visualized above each matrix). Colors represent Fisher r-to-z transformed correlations between nodes (taking into account autocorrelation and standard error), with 1-group t-test performed on all subjects with data available for a given sleep stage. Asterisks indicate significant differences ($p < 0.05$ minimum) in edge FC between stages, as determined by univariate 2-sample t-tests. Select graphical elements of this figure (FC matrices, brain images, cluster hierarchies) were generated using the FSLNets network modeling toolbox (v0.6.3; fsl.fmrib.ox.ac.uk/fsl/fslwiki/FSLNets). (A) rapid eye movement (REM) matrix below, non-REM stage 2 (NREM2) above diagonal. (B) REM below, slow wave sleep (SWS) above diagonal. SM=somato-motor, sV=striate visual, A=auditory, DAN=dorsal attention network, esV=extra-striate visual, vsV=ventral stream visual, DMN=default mode network, ECN=executive control network, l/rF-P=left/right fronto-parietal.

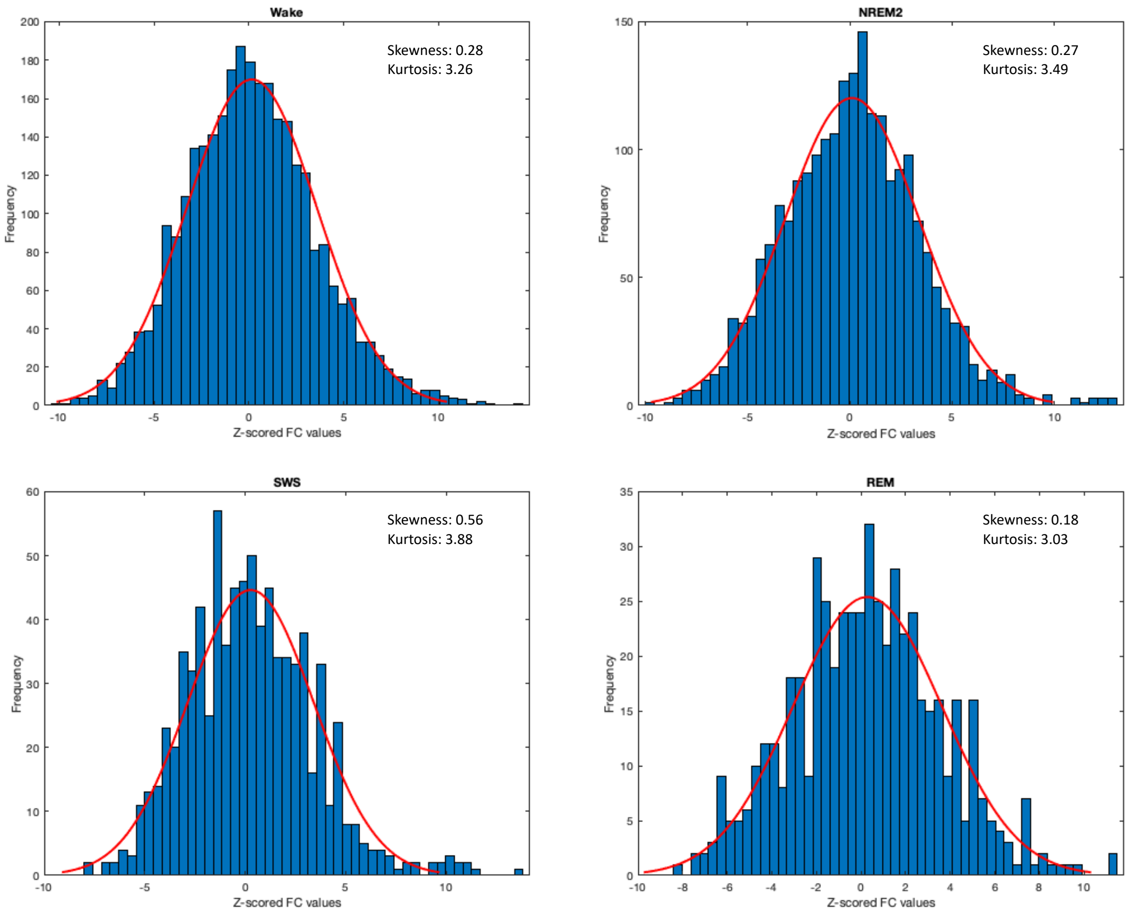

Figure S3. Histograms of functional connectivity (FC) values for each stage. Figures generated using MATLAB (R2019a; mathworks.com).

Table S1. *Sleep macrostructural data for each participant. Note that "Wake" here refers to awakenings during sleep, not separately acquired wake resting state data. TST=total sleep time*

			Wake/sleep Duration (min)					
**ID**	**Sex**	**Age**	**Wake**	**NREM1**	**NREM2**	**SWS**	**REM**	**TST**
0	F	23	21.71	3.67	20.67	70.67		95.00
1	M	22	31.44	6.00	25.02			31.02
2	F	27	25.38	5.00	25.33	8.33		38.67
4	M	23	0.95	2.78	32.00	24.33	21.67	80.78
5	M	21	11.88	2.33	20.67	19.33		42.33
6	F	23	0.03	1.00	68.00	3.33		72.33
10	M	22	12.00	17.67	26.33	7.67	19.76	71.43
12	F	28	29.19	7.33	21.67			29.00
15	F	27	42.52	3.00	23.00			26.00
17	F	33	12.42	3.00	26.33	14.67		44.00
18	F	34	3.55	6.00	55.67	3.00	26.67	91.33
23	M	27			49.15	0.33	38.33	87.81
24	M	22	43.74	6.67	22.33	21.67		50.67
26	F	22	58.68	5.00	21.00	2.33		28.33
27	M	26	54.38	2.67	20.67	0.33		23.67
28	M	27	6.02	1.33	19.67	0.67		21.67
29	F	22	21.86	5.33	8.33	39.00	3.67	56.33
30	F	20	48.30	0.67	16.33	7.67	17.00	41.67
31	M	27	79.17	6.67	8.00			14.67
33	M	23	0.93	1.00	46.99	30.67		78.66
34	F	20	36.27	10.33	17.33	0.33		28.00
35	M	28	50.05	7.33	13.33			20.67
36	F	18			11.52	13.00		24.52
37	F	20	6.23	4.67	30.00	0.33	7.00	42.00
38	F	26	10.97	6.00	26.00			32.00
41	F	21			3.33	27.67	8.27	39.27
42	M	23	32.54	7.00	14.00			21.00
43	F	20	33.60	11.00	11.33			22.33
44	F	20	24.91	18.33	8.33			26.67
**Mean**		**23.97**	**26.87**	**5.84**	**23.87**	**14.77**	**17.80**	**44.20**
**SD**		**3.83**	**20.25**	**4.38**	**14.50**	**17.17**	**10.76**	**23.84**
